# Detection of microRNA-335-5p on an Interdigitated Electrode Surface for Determination of the Severity of Abdominal Aortic Aneurysms

**DOI:** 10.1186/s11671-020-03331-y

**Published:** 2020-05-11

**Authors:** Bei Lu, Leiting Liu, Jingrui Wang, Yuan Chen, Zhijiang Li, Subash C. B. Gopinath, Thangavel Lakshmipriya, Zongwei Huo

**Affiliations:** 1 Department of Hepato-pancreato-biliary Surgery, The Affiliated Hangzhou First People’s Hospital of Zhejiang University School of Medicine, Zhejiang, 310006 Hangzhou China; 2 Department of General Thoracic and Vascular Surgery, Wuhan No. 1 Hospital, Wuhan, 430022 Hubei China; 3 Department of Endocrinology, The First People’s Hospital of Zunyi, Zunyi, 563000 Guizhou China; 4 Department of Gallbladder Pancreas and Vascular Surgery, Jingmen No. 1 People’s Hospital, Jingmen, 448000 Hubei China; 5 School of Bioprocess Engineering, Universiti Malaysia Perlis, 02600 Arau, Perlis Malaysia; 6 Institute of Nano Electronic Engineering, Universiti Malaysia Perlis, 01000 Kangar, Perlis Malaysia; 7 Department of Nuclear Medicine, Shandong Cancer Hospital and Institute, Shandong First Medical University and Shandong Academy of Medical Sciences, Jinan, 250117 Shandong China

**Keywords:** Biosensor, Noncoding RNA, Bulging aorta, Early diagnosis, Streptavidin-biotin

## Abstract

Abdominal aortic aneurysm (AAA) refers to the enlargement of the lower artery of the abdominal aorta, and identification of an early detection tool is urgently needed for diagnosis. In the current study, an interdigitated electrode (IDE) sensing surface was used to identify miRNA-335-5p, which reflects the formation of AAAs. The uniformity of the silica material was observed by 3D profilometry, and the chemically modified highly conductive surface improved the detection via the I-V mode. The targeted miRNA-335-5p was detected in a dose-dependent manner and based on linear regression and 3σ analyses, the sensitivity was determined to be 1 fM with a biotinylated probe. The high specificity was shown by discriminating the target sequence from noncomplementary and single- and triple-mismatched sequences. These outputs demonstrated the high-performance detection of miRNA-335-5p with good reproducibility for determination of the severity of AAA.

## Introduction

Abdominal aortic aneurysms (AAAs) are life-threatening diseases that are defined as an abdominal aorta with a diameter > 3 cm or larger than normal [1, 2]. This condition occurs with atherosclerosis or plaque build-up, which weakens the walls of the abdominal aorta and results in an outward bulge, similar to a balloon. Over time, the artery wall widens, and this situation is comparable to the aging of garden hoses. The pressure from the blood pumping through the aorta causes this weakened area to bulge outward, which is called an aneurysm. AAA is formed when the weakened portion of the aorta leads to complications [3–6]. AAA can lead to death caused by rupture in small aneurysms. Currently, physical examinations, computerized axial tomography angiograms, magnetic resonance imaging, and ultrasound sonography are used to diagnose this condition [7–10]. However, there are no detection methods for AAA, which is commonly identified while analyzing other health issues. This situation results in delayed identification of AAA, ultimately causing unnecessary health issues. To overcome this problem, researchers need to develop early detection methods, and one potential strategy is the development of a sensing system.

Early, rapid, and sensitive detection of disease in a quantitative manner is a vital goal for clinical diagnoses. The present biosensing platforms have met several demands and require proper laboratory settings and training. Thus, most methods are not portable, which is required for ideal point-of-care detection [11, 12]. Further, to assist doctors in decision-making in an accurate and rapid manner, an analysis of the changes in biomarker levels is highly desirable. Circulating biomarkers that are expressed in specific areas should be further investigated to diagnose AAA and follow the treatment progress. Identification of these types of circulating biomarkers will help diagnose the disease and perform patient follow-up after treatment. To fulfil these needs, this study proposes to generate sensors of appropriate biomarkers for AAA. The sensor (interdigitated electrode) proposed in this study has the potential for high-performance analysis with a wide range of biomarkers. It is a dielectrode system with alternate gaps and fingers that operate under dielectric measurements [13–15].

The biomarkers can be any biomolecules, which include DNA, RNA, proteins, carbohydrates, lipids, and their modified forms [16, 17]. In addition, researchers have proposed that different biomarkers, such as noncoding RNAs, are expressed in the cellular system, but they will not be translated into proteins [18]. Noncoding RNAs are usually not translated into proteins and generally have short sequences [18–21]. Different classes of noncoding RNAs, such as microRNAs (miRNAs), ribosomal RNAs, transfer RNAs, small nucleolar RNAs, small nuclear RNAs, telomerase RNAs, snRNAs, Xist RNAs, vault RNAs, and 7SL RNAs, have been reported. miRNAs function mainly in transcriptional and post-transcriptional regulation of gene expression and often result in gene silencing [22]. Recently, researchers described the importance of miRNAs for the prediction of AAA and reported a reduction in the expression of miRNA-335-5p in AAA patients [23]. It has been proven that the combination of clinical factors and the expression of microRNAs drastically improved the prediction of diseases and displayed increased accuracy [24]. Researchers have specifically focused on miRNA-335-5p, which displayed a significantly minimal range in individuals with fast-growing AAA [2, 23]. Furthermore, a decrease in miRNA-335-5p levels enhanced confidence of the detection of growing AAA. In other words, the negative output (higher levels) of miR-335-5p indicates the severity of AAA and minimizes laborious screening. This finding was demonstrated by Wanhainen et al. [23] and revealed that miRNAs are useful biomarkers for screening AAA and eliminating the risk of fast-growing AAA. The current study demonstrates the application of miRNA-335-5p detection by an interdigitated electrode (IDE) sensor to determine the severity of AAA in affected individuals.

## Materials and Methods

### Reagents and Biomolecules

Streptavidin, 1,1′-carbonyldiimidazole (CDI), and phosphate buffer solution (PBS) were purchased from Sigma-Aldrich, USA. Ethanolamine was purchased from Fisher Scientific, UK. All oligos were synthesized commercially from Apical Scientific Sdn. Bhd., Malaysia.

### Pattern Designing on a Chrome Mask

Initially, the pattern of the dielectric sensor was designed using AutoCAD software. The desired dimensions were a length of 7500 μm, a width of 4100 μm, 20 finger-gap pairs, a gap size of 85 μm, electrode size of 100 μm, electrode thickness of 40 nm, and a finger length of 4000 μm. The pattern was printed on a blank photomask and pasted on the surface of the chrome glass. This chrome glass was fixed under a UV exposure system for the pattern transferring procedure. A silicon dioxide substrate deposited with aluminum thin film was placed in the opposite direction against the chrome glass and exposed to UV light for 10 s. The pattern was transferred followed by the developing process using the resist developer.

### Fabrication of the Interdigitated Electrode

The surface electrode was produced via a conventional microelectronic fabrication process for fabricating the surface as follows: (i) A 4-inch wafer with existing native oxide was prepared. (ii) The wafer was cleaned using buffered oxide etch (BOE) and piranha solution to remove the native oxide and inorganic contaminants. (iii) A 2800-Å thick oxide layer was grown via an hour of wet thermal oxidation at a temperature of 1000 °C. (iv) The wafer with the oxide layer was divided into four for better handling. (v) The metal layer was deposited with l as an adhesion layer for the conductive layer. The aluminum layer (40 nm) was produced at 3 cm long with a 0.5 mm diameter via a thermal evaporator-based physical deposition method, (vi) A layer of positive photoresist was a spin coat on the substrate, (vii) The wafer was soft-baked at 90 °C for 60 s. (viii) The wafer was aligned with a chrome mask and exposed to UV light for 10 s. (ix) The wafer was rinsed in RD6 developer solution within 15 s to remove the unexposed area, resulting in the interdigitated electrode (IDE) pattern with oxide. (x) The wafer was hard-baked at 90 °C for 120 s. (xi) The wafer was etched using aqua regia to remove the exposed area of both layers. (xii) The surface pattern was produced, and the remaining photoresist was washed away using acetone. Then, 3D nanoprofilometry (Hawk 3D-profilometry, USA) analysis was carried out to observe the clear-cut image of gaps between the electrodes. Current measurements (A) were performed using Keithley 6487 with a linear sweep of 0 to 2 V at a step of 0.1 V (at 20 s).

### Surface Chemical Functionalization: Tetravalent Streptavidin-Biotin Strategy

For surface chemical functionalization, the silica surface was initially washed thoroughly with 1 M potassium hydroxide (pH 9.0) to activate the surface. This surface was further modified by CDI (0.5 M) with an incubation period of 1 h to react with 100 nM streptavidin (for 1 h), diluted from the original stock in 10 mM phosphate-buffered saline (PBS; pH 7.4). Following this step, the unreacted spaces were blocked using 1 M ethanolamine (for 1 h). Then, the tetravalent streptavidin was reacted with 1 μM of biotinylated probe miRNA-335-5p (for 10 min) at room temperature. Upon completion of each modification, the surface was thoroughly washed with PBS unless otherwise stated.

### Designing the Probe and Target Sequences from miRNA-335-5p

The full-length miRNA-335-5p (5′-UGUUUUGAGCGGGGGUCAAGAGCAAUAACGAAAAAUGUUUGUCAUAAACCGUUUUUCAUUAUUGCUCCUGACCUCCUCUCAUUUGCUAUAUUCA-3′) with the accession code MIMAT0000765, has been collected from the data bank. The desired region for this study as the target is 5′-UCAAGAGCAAUAACGAAAAAUGU-3′. To capture the target miRNA-335-5p region, the probe was designed as 5′-ACAUUUUUCGUUAUUGCUCUUG-3′. At the 3′-end, biotin was tagged to interact with streptavidin immobilized on the sensing surface. The secondary structure of the full-length miRNA-335-5p was predicted by online mfold software (http://unafold.rna.albany.edu/?q=mfold).

### Probe-Target Interactions: miRNA-335-5p Sequences

Upon binding of the biotinylated probe to the streptavidin surface as detailed above, the target miRNA-335-5p sequence interacted (for 10 min) at ambient room temperature from the low-femtomolar to the low-nanomolar concentrations. These concentrations ranged from 1 fM to 1 nM with 10-fold serial dilutions in PBS. The attachments were tested independently from lower to higher concentrations, and upon duplex formation, the surface was washed with 5-fold volumes of PBS, and measurements were taken.

### I-V Measurements

The electrical characterizations were performed by I-V measurements using a Keithley 6487 Picoammeter with a voltage/current supply. The measurements were recorded at the abovementioned volts on the bare surface followed by on each surface chemical modification. Similarly, for the interactive analysis between the probe and target miRNA-335-5p at each concentration, the measurement was performed after the washing step with 10 reaction volumes. All modifications, interactions and measurements were maintained under wet conditions unless otherwise stated.

## Results and Discussion

### Fabrication of the IDE Sensor and Surface Analysis

**Fig. 1:**
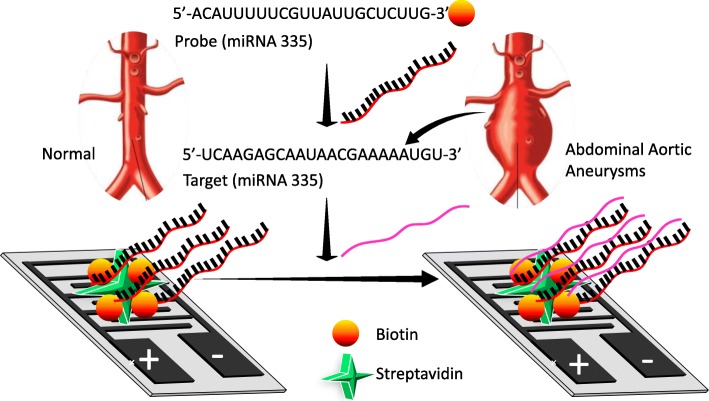
Representation of the detection of miRNA-335-5p on an IDE. The designed probe and target are shown along with the duplex formation on the IDE surface

**Fig. 2:**
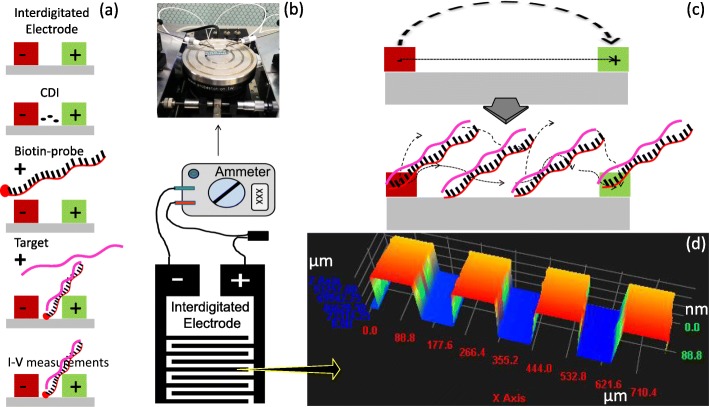
Surface measurements on an IDE. **a** Measurement steps. **b** Measuring mode. The IDE pattern and the connecting system are shown. A dielectric measurement is also displayed. **c** Surface mechanism during measurement. A dipole moment is shown. **d** The 3D profilometry profile of the fabricated IDE

The current study used miRNA-335-5p as the tool to elucidate the severity of AAA by interactive analysis on an interdigitated electrode (IDE) sensor (Fig. 1). For the interactive analysis with the probe and the target designed from the full length of miRNA-335-5p, an IDE surface was designed and fabricated using conventional photolithographic techniques. Step-by-step surface modifications were carried out and measured by an ammeter (Fig. 2b, c). The mask alignment designed for the fabrication of IDE and the uniformity were confirmed by high-resolution 3D nanoprofilometry analysis. This observation confirmed that the fabrication and the distance between each finger were well aligned (Fig. 2d). IDEs have an electrode size of ~100 μm with a spacing of ~85 μm, and a large intercalated network with millions of biomarkers in series/parallel is formed. To ensure the success of the fabricated IDEs, we used different bare devices to determine the reproducibility under the dielectric system. This system measures the molecular vibrations between the dielectrodes caused by the dipole moment (Fig. 2c). This dielectric IDE is a well-accepted system that has been widely used for different biomolecular and chemical analyses [15, 25–28]. In this study, all the surface chemical modifications and interactive analyses were measured by the I-V current mode.

### High-Density Surface Molecular Assembly: Streptavidin-Biotin Strategy

High-density molecular attachments on the sensing surface or biochips have been shown to be crucial and are highly dependent on surface functionalization [29, 30]. This study aimed to determine the appropriate surface needed for a high number of molecular accommodations, which is mandatory for gene expression, discovery, and molecular analysis. For these potential applications, the generation of analytical system surfaces requires the proper coupling of molecules to immobilize them in appropriate formats. These material surfaces were studied to investigate the surface interactive mechanism, as they are cheap and provide varied degrees of packing densities, morphology, and thickness. This finding will facilitate the proper attachment of molecules and prevent the loss of biomolecular activity. Furthermore, the right orientation of biomolecular immobilization is retained with proper secondary structural confirmation.

**Fig. 3:**
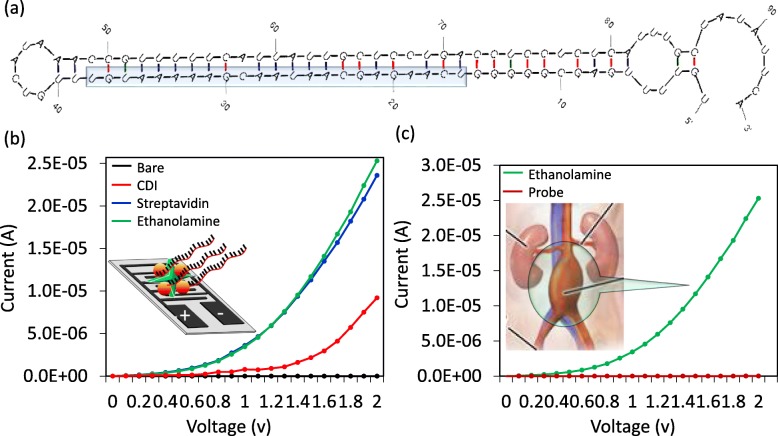
**a** Predicted secondary structure of miRNA-335-5p. The full-length miRNA-335-5p is displayed with the stem and loop regions. The selected target region for the interactive analysis is indicated. **b** I-V measurements on the IDE for the surface chemical and biological modifications (black circle, bare; red circle, CDI; dark blue circle, streptavidin; green circle, ethanolamine). The figure inset is shown diagrammatically. **c** I-V measurements on the IDE for the probe attachment. (green circle, ethanolamine; dark red circle, probe). The image for the AAA complication is shown as the figure inset

**Fig. 4:**
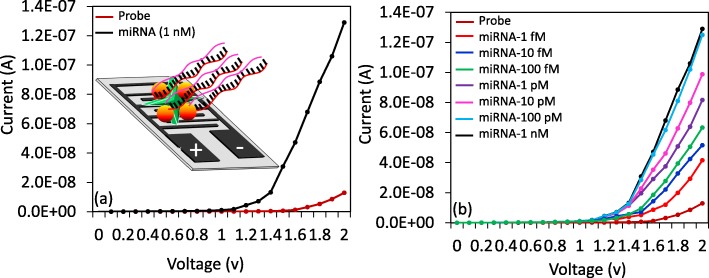
Interactive analysis of the probe and target. **a** Higher-dose interaction of the probe and miRNA-335-5p (target). (dark red circle, probe; black circle, target). **b** Dose-dependent analysis of miRNA-335-5p. Testing from the lower femtomolar to the lower nanomolar range. (dark red circle, probe; orange circle, 1 fM; blue circle, 10 fM; green circle, 100 fM; purple circle, 1 pM; pink circle, 10 pM; light blue circle, 100 pM; black circle, 1 nM)

To achieve high-density molecules on the surface, we initially thoroughly washed the silica surface with alkali solution and modified it by CDI. To capture high levels of biotinylation probes on the CDI surface, we immobilized the probe with streptavidin. Streptavidin is a tetravalent protein with four sites to bind biotin and is considered a high-affinity molecule in the biological sciences [31, 32]. The surface of IDE had a current level of 2.08E− 10 A, and after the CDI attachment, it was increased to 9.2E− 06 A. This finding clearly indicates that the surface of the IDE modified by CDI is appropriate for our analyses. When streptavidin was added, the current level was further increased to 2.36E− 05 A. Using this strategy, we immobilized a higher number of biotinylated probes designed from full-length miRNA-335-5p on the IDE surface (Fig. 3a). Biotin interactions were carried out after the blocking step on the IDE surface to mask the unreacted areas. With each surface modification, the changes in the current were determined (Fig. 3b). As shown in Fig. 3c, after addition of ethanolamine, the current changes were 2.53E− 05 A, and when 1 nM of biotinylated capture sequence was added, the level of the current was decreased to 1.29E− 08 A. This change in the current confirmed the immobilization of the capture probe on the IDE sensing surface. Generally, a single-stranded oligonucleotide carries a more negative charge with the exposed 5-carbonated phosphate backbone (probe), whereas the net charge of ethanolamine is neutral, as stated by the supplier. This large difference in charge resulted in an apparent variation in the measured current, as shown in Fig. 3c. Further, this parameter was reverted again upon duplex formation with the target strand. This finding is due to the reduction in the exposure of the phosphate backbone, and the additional positive charges originate from the nucleobases in the miRNA strand (Fig. 4a).

### Dose-Dependent Interactive Analysis on the IDE

Upon attachment of the biotinylated probe on the sensing surface, the target interactive analysis was performed with different doses. This validation was performed from the low-femtomolar to the low-nanomolar range. To ensure this range, we performed the preliminary analysis with 1 nM of target miRNA-335-5p sequences. After addition of 1 nM of the target miRNA-335-5p sequence on the capture probe-modified surface, the current level was changed from 1.29 E− 08 A to 1.29E− 07 A (Fig. 4a); this result indicated that hybridization occurred between the capture probe and the target sequence. After this confirmation, to determine the limit of detection, we titrated the target sequence from 1 fM to 1 nM, which dropped independently on the capture probe-modified surfaces. The current changes were detected before and after immobilization. The differences in current for the 1 fM, 10 fM, 100 fM, 1 pM, 10 pM, 100 pM, and 1 nM hybridizations were 2.87, 3.87, 5.04, 6.88, 8.59, 11.21, and 11.61E− 08 A, respectively. Based on the obtained results, clear dose-dependent current changes were detected in all the tested concentrations of the target sequence (Fig. 4b).

### High Analytical Performance: Sensitivity, Specificity, and Reproducibility

**Fig. 5:**
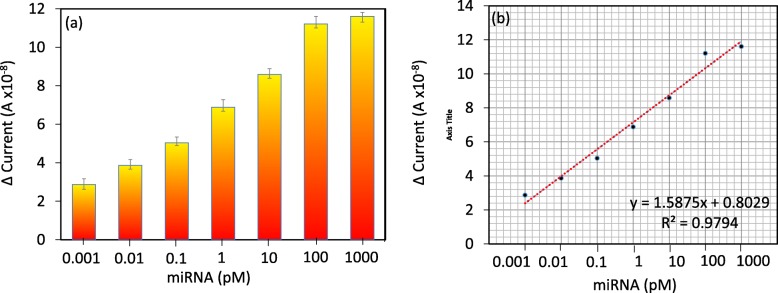
Dose-dependency. **a** Dose-dependent interaction of miRNA-335-5p with the probe. **b** Linear regression analysis with different concentrations of miRNA-335-5p. LOD was considered the lowest concentration of an analyte against the background signal (S/N = 3:1)

**Fig. 6:**
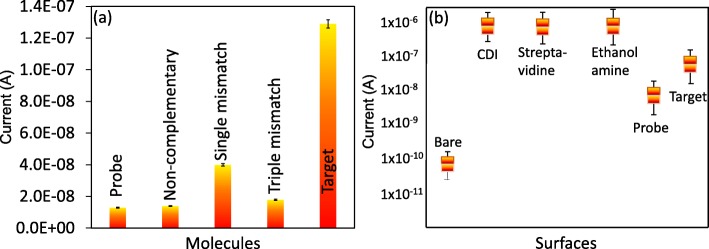
High-performance analysis. **a** Specificity analysis. Target sequences were discriminated from the noncomplementary and single- and triple-mismatched sequences. **b** Reproducibility test. Averaged values are displayed with triplicate experiments

Based on the above results with concentration-dependent increments, linear regression analysis was performed. The sensitivity of the detection was estimated using the 3σ calculation, and it fell at 1 fM (Fig. 5a, b). The concentration was less than 1 fM, showing the background signal and lower values. Further, specificity analysis was performed to discriminate the target sequences from the noncomplementary and single- and triple-mismatched sequences. It was apparent that the designed probe showed a stronger reaction with the perfect miRNA-335-5p complementary sequences than the other sequences (Fig. 6a). A single-mismatched sequence resulted in a higher current response than the noncomplementary and triple-mismatched sequences due to the partial duplex formation by the single-mismatched sequence on the probe sequence. Reproducibility analyses were carried out with all chemical and biological modifications on the IDE surface in triplicate, and the data were averaged. The averaged data indicate that there were no significant changes when the different experiments were performed (Fig. 6b). Further, to support the performance of the IDE, the characteristics of currently available methods were compared (Table 1).

**Table 1:** Comparison with the current detection methods

Method	Measurement	Measurement mode	Accuracy	Accuracy mode	Ref.
Interdigitated electrode	0–2 V (current-volt)	Immunosensing	At low picomolar	Dose-dependent analysis	[33]
Ultrasonic transducer	40 kHz	Oscillator	2 to 400 cm	Distance traveled	[34]
Telemetric pressure	95–130 mmHg	Intra-aortic pressure	10 cm	Transmission range	[35]
Telemetric pressure	133 kHz	Operating frequency	120 mm	Transmission range	[36]
Wireless pressure	10–20 MHz	Operating frequency	40.27 kHz/mmHg	Resonant frequency	[37]
Interdigitated electrode microwave	24 GHz	Operating frequency	200 MHz and 4 GHz	Frequency	[38]
Interdigitated electrode	0–2 V (current-volt)	Duplex formation	At low femtomolar	Dose-dependent analysis	Current work

## Conclusions

Development of an analytical method using biological biomarkers aids in diagnosing AAA during physical examinations. In the current research, miRNA-335-5p-mediated detection for predicting the severity of AAA was developed, as miRNA-335-5p was found to be expressed with AAA. The output of the generated IDE detection displayed lower to higher detection levels to facilitate the prediction of the severity of AAA. The lower detection level was 1 fM with high specificity and reproducibility for the high-performance analysis. The tetravalent streptavidin-biotin method used in this research yielded a good output and can be utilized for other biomarker analyses.

## Data Availability

All data are fully available without restriction.

## Abbreviations

AAA: Abdominal aortic aneurysm
**CDI**: **1,1′-Carbonyldiimidazole**
PBS: Phosphate buffer solution
IDE: Interdigitated electrode
UV: Ultraviolet
